# Positively Charged NF Membranes with Co-Enhanced Donnan and Size-Sieving Effects Fabricated Toward Efficient Li^+^/Mg^2+^ Separation

**DOI:** 10.3390/membranes16080270

**Published:** 2026-08-13

**Authors:** Yu-Tong Yin, Rui Jia, Zhen-Liang Xu, Sen Wang, Rui Han

**Affiliations:** 1 State Key Laboratory of Chemical Engineering and Low-Carbon Technology, Membrane Science and Engineering R&D Lab, Chemical Engineering Research Center, School of Chemical Engineering, East China University of Science and Technology, 130 Meilong Road, Shanghai 200237, China; 2 Shanghai Electronic Chemicals Innovation Institute, East China University of Science and Technology, Shanghai 200237, China

**Keywords:** surfactant, Interlayer, Nanofiltration membrane, Interfacial polymerization, Li^+^/Mg^2+^ separation

## Abstract

Extracting lithium from salt-lake brines boasts distinct advantages including low production cost, low energy consumption and low environmental risks, and will serve as a primary supply source of lithium salts in the future. This trend raises higher demands for the efficiency and cost-effectiveness of lithium extraction technologies. Nanofiltration (NF) membranes, renowned for their superior discrimination between monovalent and divalent ions, have been extensively utilized to obtain Li^+^ from Mg^2+^-rich saline brines. In this study, positively charged NF membranes aimed at Li^+^/Mg^2+^ fractionation were fabricated via surfactant-interlayer-assisted interfacial polymerization (SIAIP). Catechol (CA) and polyethyleneimine (PEI) were utilized to construct the CA/PEI interlayer, and oil-phase dodecyl phosphate (DDP) was used as an additive for interfacial polymerization (IP). The strongly bonded CA/PEI nanoaggregates improved interlayer stability and preserved the positive charge of the double-layer membrane. DDP adsorbed piperazine (PIP) at the two-phase interface through electrostatic interactions, accelerating PIP diffusion and forming a thick polyamide (PA) layer with uniform pores. The combination of CA/PEI interlayer and DDP synergistically enhanced size-sieving and Donnan effects. With MgCl_2_ and LiCl rejections of 97.9% and 36.2% respectively, the optimized membrane shows superior selectivity for Li^+^ over Mg^2+^. Moreover, the membrane exhibited weak electrostatic screening and concentration polarization, showing excellent operational stability under varied Mg^2+^-Li^+^ ratios and feed concentrations.

## 1. Introduction

For contemporary energy industry, lithium serves as a strategically vital mineral commodity [1,2,3], with wide applications in industrial production [4], aerospace, pharmaceuticals [5], and other fields. Salt lake brines represent abundant lithium reserves [6], yet the coexisting sodium, magnesium, and calcium cations hinder the production of high-purity lithium [7]. Given the similar chemical characteristics and hydration radii (3.82 Å for Li^+^ and 4.28 Å for Mg^2+^), high magnesium content further increases the difficulty of lithium purification [8]. Hence, Li^+^/Mg^2+^ separation is a key process for salt-lake lithium extraction. Operating under pressure driving force, nanofiltration (NF) membranes can discriminate monovalent cations from multivalent ionic species through the synergy among steric hindrance, Donnan repulsion and dielectric exclusion [9]. With low energy consumption and tunable performance via simple modification strategies, NF has broad industrial application prospects and is promising for Li^+^/Mg^2+^ fractionation in saline lake brines [10]. Thin-film composite (TFC) NF membranes serve as core materials for selective ion separation. Their separation capability originates from the coupling of size-sieving and Donnan repulsion, enabling differential transmission between monovalent and multivalent ions. Owing to this feature, TFC-NF membranes have aroused extensive attention in salt-lake lithium extraction [11,12]. Precisely tuning the pore size, pore size distribution, hydrophilicity and surface charge density of the polyamide (PA) selective layer has become a prevalent strategy to improve the ion fractionation capability of TFC NF membranes. For lithium extraction from high-magnesium salt-lake brines, synergistic optimization of these membrane properties can integrate size-sieving and Donnan exclusion effects, realizing efficient discrimination between Li^+^ and Mg^2+^ [13,14,15].

Positively charged NF membranes are specifically tailored to amplify the Donnan exclusion effect. These membranes generate strong electrostatic repulsion against divalent magnesium ions while permitting monovalent lithium ions to permeate, realizing efficient Li^+^/Mg^2+^ separation [16,17,18]. Zhao et al. [19] synthesized cationic polyamine NF membranes through interfacial polymerization (IP) between polyethyleneimine (PEI) and 1,3,5-tris(bromomethyl)benzene (TBB). The formed selective layer had a thickness of around 95 nm and an effective pore size of 0.65 nm and displayed a strong positive surface charge, with divalent salt rejection above 90%. Zhang et al. [20] adopted ethylenediamine-terminated PEI (EDA@PEI) as water-phase monomer to undergo IP with trimesoyl chloride (TMC). Remarkable improvements in crosslinking degree and zeta potential were observed for the synthesized PA layer, which exhibited outstanding separation performance. Compared with conventional negatively charged NF membranes, positively charged membranes exhibit unique superiority for salt-lake lithium extraction. Negatively charged membranes inevitably adsorb Mg^2+^ and Ca^2+^ in brine via electrostatic attraction, causing severe inorganic scaling, irreversible fouling, and deteriorated Li/Mg selectivity. In contrast, positively charged membranes effectively repel multivalent cations through the Donnan exclusion effect, significantly inhibiting cation deposition and scaling while maintaining high lithium-magnesium separation efficiency [21,22]. This distinctive anti-cation-fouling capability renders positively charged NF membranes more adaptable to high-magnesium salt-lake brines.

Tuning pore size distribution to reinforce size exclusion effect is another feasible route for NF membranes to distinguish Mg^2+^ and Li^+^ via size-based screening. Introducing surfactants to accelerate monomer mass transfer across the water–oil interface is an established tactic to construct homogeneous sub-nanometer pore channels. The research group led by Jin [23] incorporated sodium dodecyl sulfate (SDS) into aqueous phase. The diffusion of PIP monomer was enhanced due to amphipathic self-assembled network that developed at the interfacial boundary, yielding a PA separation layer with evenly distributed sub-angstrom pores. In their subsequent work [24], oil-soluble dodecyl phosphate (DDP) was introduced into organic phase. The resultant NF membranes exhibited narrowed pore size distribution ranging from 0.32 to 0.80 nm, which fell exactly between the Stokes diameters of Mg^2+^ and Li^+^. Wang et al. [25] adopted 1,4,7,10-tetraazacyclododecane (cyclen), a novel macrocyclic polyamine, as the aqueous monomer. SDS was added as a phase transfer catalyst to alleviate mass transfer limitations associated with the large size of cyclen molecules, thereby reducing imperfections of PA layer. The as-fabricated m-SARIP membranes delivered high permeance and outstanding perm-selectivity toward monovalent and multivalent cations.

Based on the literature review, synergistic regulation of surfactants and interlayers provides a promising route to fabricate NF membranes featuring simultaneously strengthened Donnan and size-sieving effects. Accordingly, the surfactant-interlayer-assisted interfacial polymerization (SIAIP) strategy has been developed in previous studies [26]. In this work, to achieve efficient Li^+^/Mg^2+^ separation, we take advantage of the coordination effect between surfactant additives and substrate interlayers for fabricating dual-charged TFC NF membranes. During IP, the surfactant DDP is incorporated to adjust monomer transport behavior on the oil–water interface, which shrinks the average pore dimension and reinforces the steric sieving capacity. In the subsequent step, catechol (CA) is adopted to build a positively charged CA/PEI intermediate layer. Powerful intermolecular interactions (covalent bonds, hydrogen bonding and π-π stacking) induce self-assembly of polyphenol and polyamine components into evenly distributed nano-adhesive agglomerates. These aggregates greatly improve the structural stability of the PEI interlayer and ensure membrane surface positively charged [27]. The joint modulation of CA/PEI interlayer and DDP enables simultaneous enhancement of size-sieving and Donnan effects.

## 2. Experiments

### 2.1. Materials

Polyethersulfone (PES) substrates (0.22 μm) were provided by Longjin Membrane Technology Co., Ltd., Nantong, China. CA and mono-n-dodecyl phosphate (DDP, ≥99.0%) were supplied by Shanghai Macklin Biochemical Technology Co., Ltd., Shanghai, China. PEI were supplied by Shanghai Aladdin Biochemical Technology Co., Ltd., Shanghai, China. PIP (≥99.5%) and TMC (≥99.5%) were provided by Sigma-Aldrich, St. Louis, MO, USA. Sodium periodate (NaIO_4_, ≥99.5%) was supplied by Shanghai Titan Technology Co., Ltd., Shanghai, China. Sinopharm Chemical Reagent Co., Ltd., Shanghai, China supplied lithium chloride (LiCl, ≥99.5%), magnesium chloride (MgCl_2_, ≥99.5%), sodium sulfate (Na_2_SO_4_, ≥99.5%), ethylene glycol (EG, ≥99.0%), n-hexane, (≥97.0%), and polyethylene glycols (PEGs) with different molecular weights.

### 2.2. Preparation of the Interlayer

**Figure 1:**
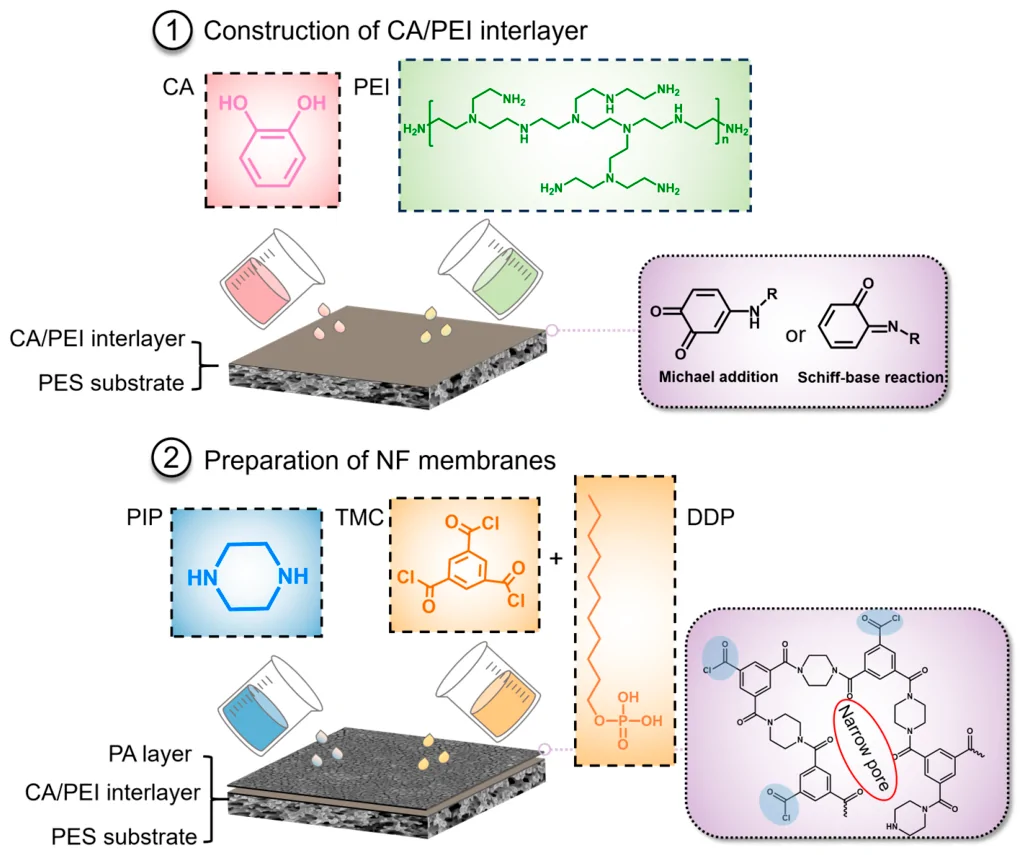
Schematic of SIAIP membrane preparation.

As shown in Figure 1, the fabrication process of CA/PEI interlayer was modified based on reported protocols [28]. A solution of 0.3 g PEI in 50 mL deionized (DI) water was prepared as a reserve. Separately, 1 g CA was dissolved in 50 mL DI water, followed by adding 0.05 g NaIO_4_. The mixture was stirred thoroughly to obtain quinone compounds generated from the oxidation of CA. Finally, 100 mL CA/PEI mixed liquor was prepared by slowly pouring the PEI solution into CA solution under high-speed stirring.

The PES substrate membrane, which had been soaked in DI water and air-dried, was fixed with stainless steel clamps. A 20 mL aliquot of CA/PEI solution was applied to its surface for a duration of 20 min. Upon removal of the excess liquid, the sample was washed and dried for future use. The original and modified substrates were labeled as PES and PES-CA/PEI, respectively.

### 2.3. Fabrication of NF Membranes

The PIP aqueous solution (0.5 wt%) was deposited onto PES substrate, kept there for 2 min, and finally the extra fluid was discarded. Then an air-knife was used to blow the membrane until no visible water traces remained. Subsequently, the membrane was soaked in a bath of 0.05 wt% TMC dissolved in n-hexane for 30 s to implement IP, with excess organic solvent removed once the polymerization terminated. After air-drying, the membrane was thermally treated in an oven at 60 °C for 2 min. The resultant membrane prepared via traditional interfacial polymerization was designated as t-IP membrane.

DDP was added into the TMC/n-hexane solution to a concentration of 0.15 mM, while all other reaction parameters remained unchanged. The obtained membrane was defined as the surfactant-assisted interfacial polymerization (SAIP) membrane. The IP process was subsequently performed on the PES-CA/PEI support, with 0.15 mM DDP added to organic phase. The corresponding membrane was denoted as the SIAIP membrane. Table 1 compiles the detailed fabrication conditions for all three membrane types.

### 2.4. Characterization

Scanning electron microscopy (SEM, Nova Nano SEM450, Hillsboro, OR, USA) and atomic force microscopy (AFM, Bruker Dimension Icon, Karlsruhe, Germany) were adopted to observe surface microstructure and quantify membrane roughness. The chemical constitution of all as-prepared membranes was analyzed via Fourier transform infrared (FTIR) Spectroscopy (Thermo Scientific Nicolet iS20, Waltham, MA, USA) and X-ray photoelectron spectroscopy (XPS, Thermo Scientific K-Alpha+, Waltham, MA, USA). The hydrophilicity and surface potential were evaluated using a water contact angle meter (WCA, JC2000A, Zhong Chen, Shanghai, China) and a potential analyzer (SurPASS 3, Anton Paar, Graz, Austria). The relevant characterization methods are detailed in Supporting Information Section S1.

### 2.5. Membrane Performance Test

The permeation and separation performance of the NF membrane was tested using a laboratory-made cross-flow filtration device with the test area of 22.05 cm^2^ and the cross-flow velocity of 22 cm·s^−1^. The temperature was kept at 25 °C, and the membrane should be pre-pressurized at 5 bar for 30 min before the test. The concentration of the three inorganic salt (MgCl_2_, LiCl, and Na_2_SO_4_) solutions was 1000 ppm, and the salt concentrations in the feed and permeate solutions were analyzed by a conductivity meter (DDS-11A, Lei Ci, Shanghai, China). For accuracy, the mean and error bars were calculated using data from at least three sets of parallel experiments under the identical conditions. The detailed method for membrane performance testing is provided in Supporting Information Section S1.

## 3. Results and Discussion

### 3.1. Structural Properties of CA/PEI Interlayer

**Figure 2:**
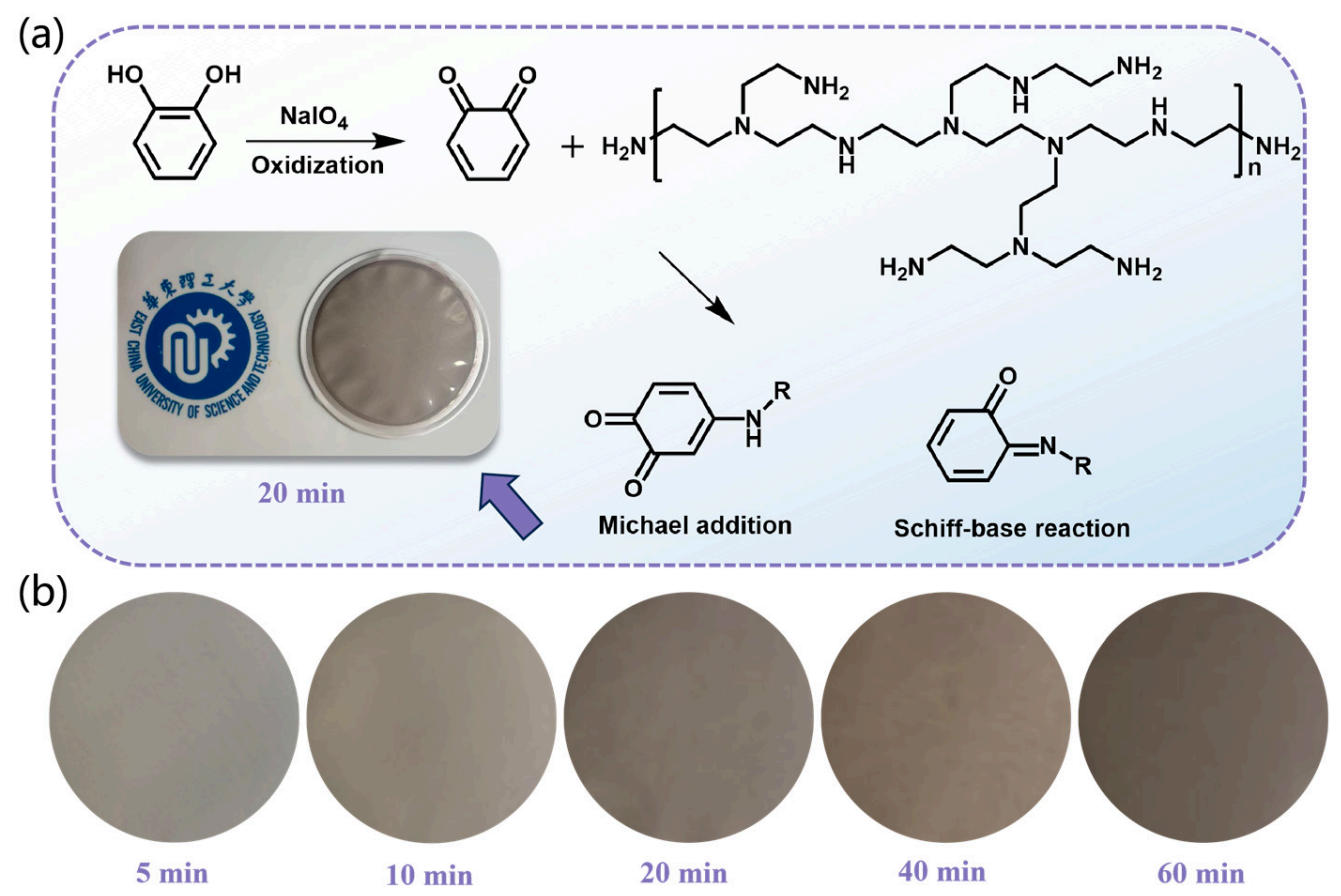
Schematic illustration of (**a**) reaction mechanism between CA and PEI, (**b**) substrate surface under different deposition times.

Figure 2a illustrates the formation mechanism of CA/PEI interlayer. CA is oxidized to quinone structures by NaIO_4_. Subsequently, CA binds with amino groups via Michael addition reaction or Schiff base reaction, ultimately forming a brown interlayer composed of nano-sized CA/PEI adhesive aggregates. This reaction mechanism has been validated in previous studies [29,30]. Figure 2b shows the surface morphology of substrate membranes at different deposition durations. The membrane surface gradually changes from light brown to dark brown as the deposition time increases. This suggests that extending the deposition time promotes greater adhesion of CA/PEI aggregates onto the substrate, ultimately resulting in a thicker interlayer. With the DDP dosage fixed at 0.15 mM, the optimal conditions for interlayer fabrication were identified as follows: CA concentration of 1 wt%, PEI concentration of 0.3 wt%, and an interlayer deposition time of 20 min. The detailed investigation on the optimal preparation conditions for the CA/PEI interlayer is presented in Supporting Information Section S2 and Figures S1–S3.

**Figure 3:**
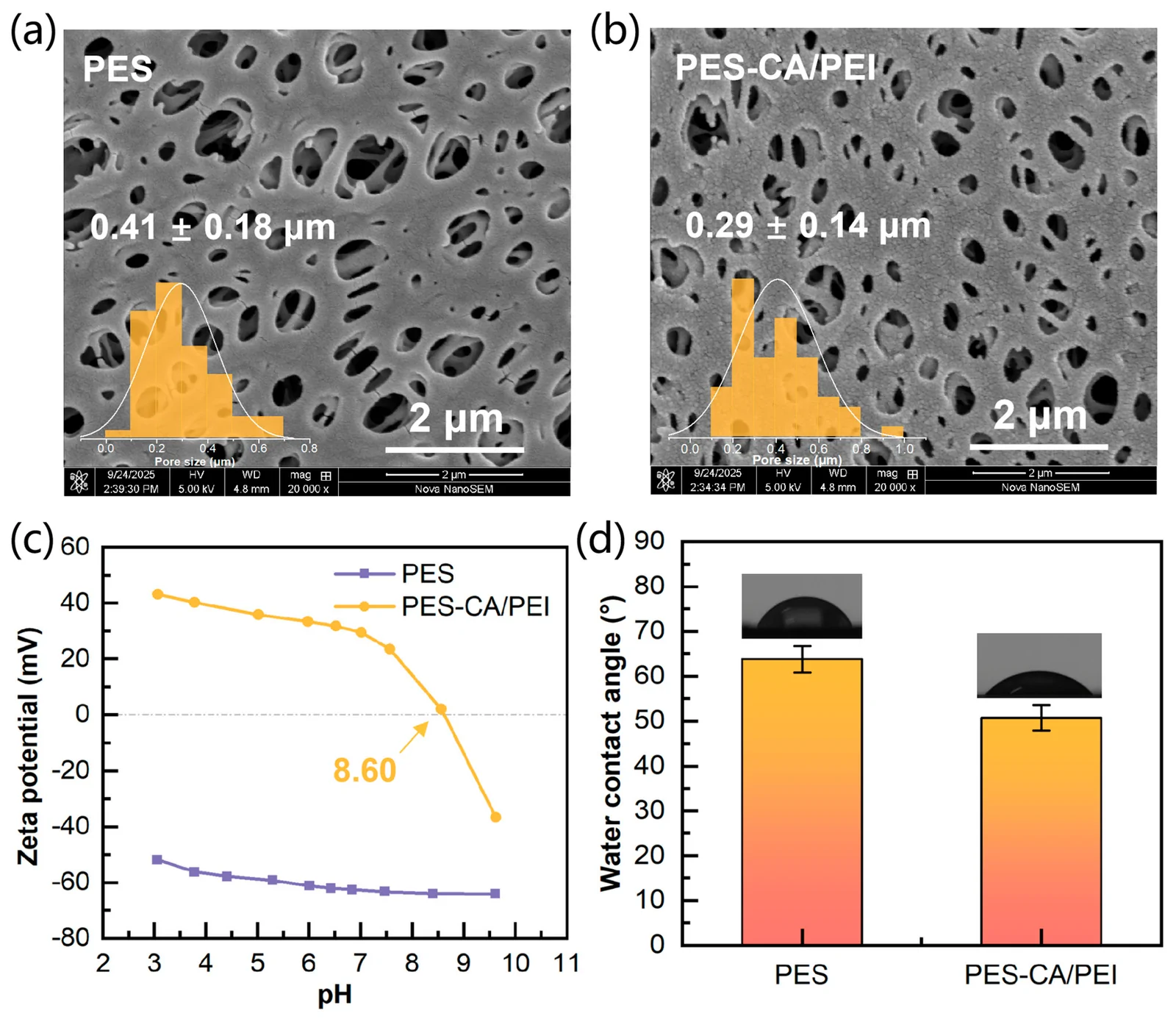
The (**a**,**b**) SEM images, (**c**) Zeta potential, and (**d**) WCA of PES and PES-CA/PEI.

The surface morphologies of the pristine PES substrate and the PES-CA/PEI membrane fabricated under the optimal interlayer preparation conditions are shown in Figure 3a,b. The pristine PES substrate is characterized by irregular pore dimensions and a disordered distribution, possessing an average pore diameter of roughly 0.41 µm. Conversely, the average surface pore size of PES-CA/PEI membrane after modification is reduced to 0.29 µm, with uniformly dispersed nanoparticles clearly visible on the surface. These observations verify the successful immobilization of the nano-sized CA/PEI interlayer onto the substrate. Figure 3c,d are the surface potential and WCA measurements obtained for the membranes before and after modification. After introducing the CA/PEI interlayer, the surface potential changes dramatically: the strongly negative surface charge of the pristine PES substrate is converted to positive charge, with an isoelectric point at pH 8.6. The improved surface charge is attributed to abundant amino groups introduced by the CA/PEI coating. Protonation imparts a strong positive charge to these amino groups, greatly boosting the surface’s positive charge density. Additionally, the products generated from phenol-amine reactions introduce abundant hydrophilic functional groups, specifically phenolic hydroxyls and amines. Accordingly, the WCA declines from 63.8° to 50.7° after interlayer modification, demonstrating that the CA/PEI interlayer can effectively enhance surface hydrophilicity of the substrate.

### 3.2. Chemical Characterization of Membrane Surfaces

**Figure 4:**
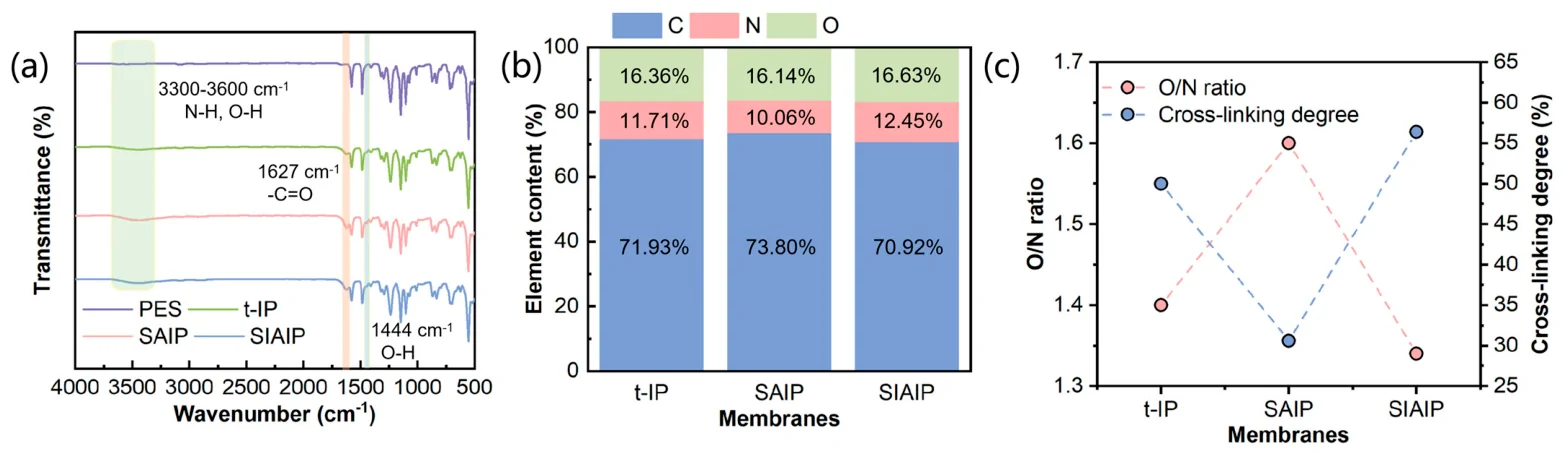
The (**a**) FTIR, (**b**) chemical components, and (**c**) O/N ratio and crosslinking degree of prepared membranes.

Figure 4a displays the FTIR spectra recorded for the three NF membranes. Characteristic absorption bands are observed at 3300–3600 cm^−1^ and 1627 cm^−1^ for all samples. The peaks stem from both the -–OH and –NH stretching vibrations associated with carboxyl residues or unreacted PIP monomers, and the –N–C=O functionalities that emerge from the IP reaction. These characteristic bands are absent in the pristine PES substrate, which strongly verifies that PA functional layers have been successfully fabricated on the surface of three NF membranes via IP reaction [31]. Compared with the t-IP membrane, the SAIP and SIAIP membranes exhibit higher absorption intensity in the range of 3300–3600 cm^−1^. This phenomenon is attributed to the addition of DDP, which facilitates the interfacial diffusion of amine monomers at the oil–water interface, consequently raising the amount of unreacted -NH moieties present on membrane surface [24].

Surface elemental compositions, calculated O/N atomic ratios and crosslinking degrees of membranes are shown in Figure 4b and Figure 4c, respectively. Detailed calculation procedures are available in Supporting Information Section S3 [32] and Table S1. The crosslinking degree is defined as the proportion of crosslinked network PA in total PA (crosslinked network plus linear structures). It is generally accepted that a higher crosslinking degree corresponds to a denser PA separation layer, while a lower value indicates a looser structure [33]. While the t-IP membrane exhibits a crosslinking degree of 50.0%, the SAIP counterpart shows a sharp decrease to 30.7% due to incorporation of DDP into organic phase. This result reveals that while DDP efficiently controls PIP monomer migration at the interface, it impedes the development of a highly crosslinked PA network in the upper layer. Conversely, incorporating a CA/PEI interlayer significantly elevates the crosslinking degree of the SIAIP membrane to 56.4%. This highlights that the synergistic interaction between DDP and the CA/PEI interlayer facilitates the creation of dense, defect-free PA separation layers.

**Figure 5:**
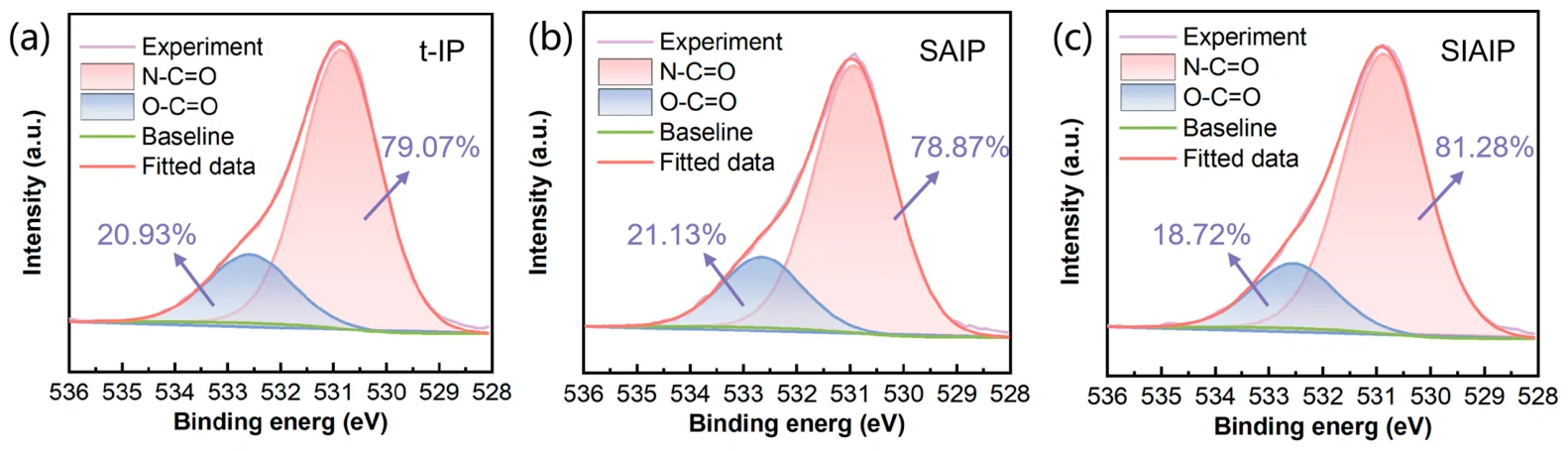
O 1s spectra of (**a**) t-IP, (**b**) SAIP, and (**c**) SIAIP membranes.

Fitted high-resolution O 1s XPS spectra of PA layers are displayed in Figure 5a–c, revealing two distinct peaks assigned to O–C=O and N–C=O functionalities. The O–C=O species originate from hydrolysis of acyl chloride groups present in TMC monomers, whereas the N–C=O moieties are generated via IP [34]. For SAIP membrane (with DDP added to the organic phase), the relative N–C=O abundance on its surface drops moderately to 78.87% compared with that of t-IP membrane. In contrast, SIAIP membrane, which incorporates a CA/PEI interlayer, exhibits a substantial rise in N–C=O content to 81.28%. This trend closely parallels the variation in crosslinking degree of the PA layers discussed above, further confirming the correlation between crosslinking degree and polymerization extent. Notably, the SIAIP membrane exhibits the lowest relative content of O–C=O at only 18.72%. This phenomenon indicates that the synergistic effect between DDP surfactant and CA/PEI interlayer optimizes the interfacial mass transfer of PIP monomers and promotes their sufficient reaction with TMC. Accordingly, the residual unreacted TMC at the interface is reduced, thereby hindering acyl chloride group hydrolysis and cutting down the generation of byproducts with O–C=O.

### 3.3. Morphological Characterization of Membranes

**Figure 6:**
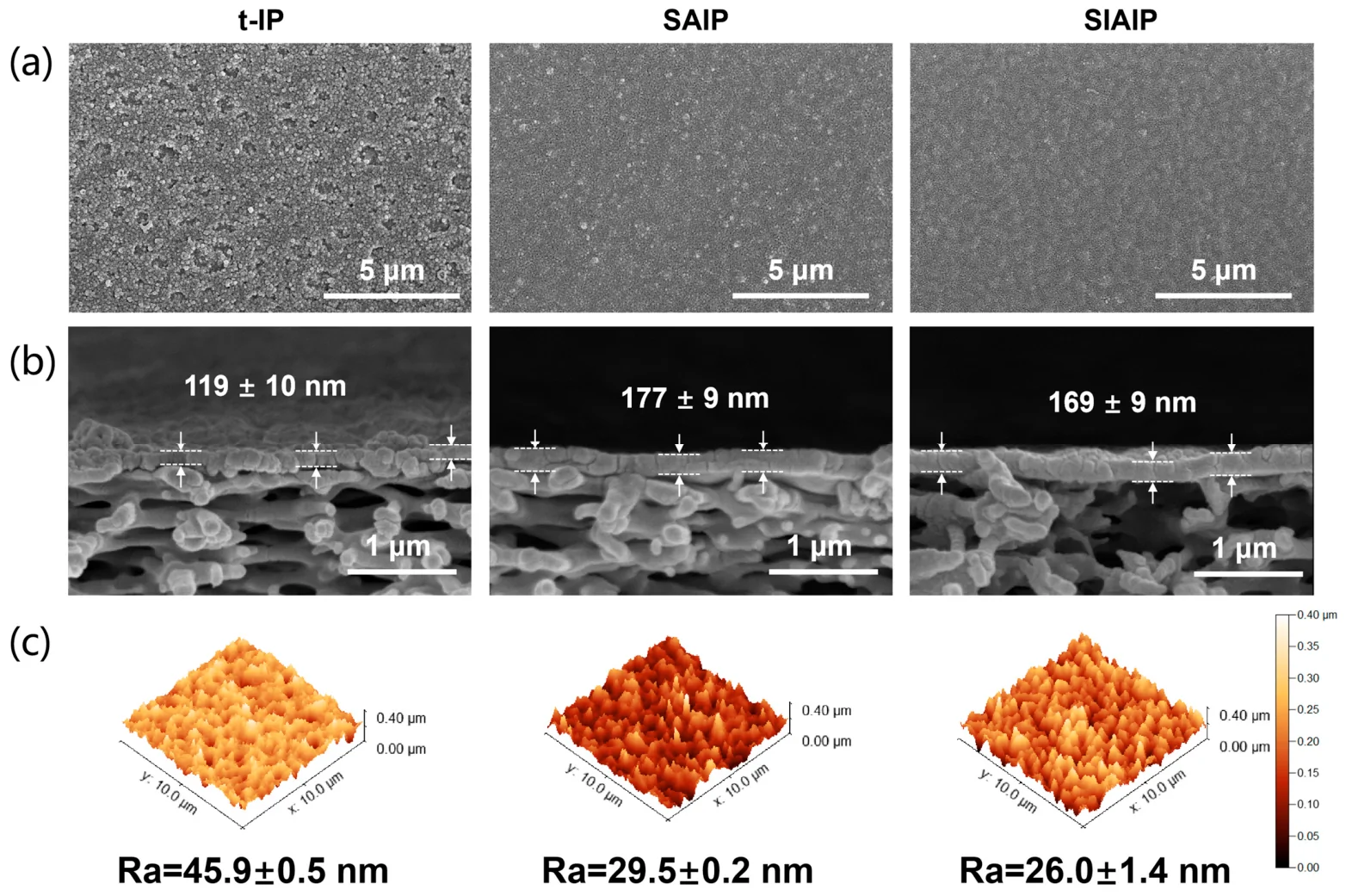
SEM images of (**a**) surface morphology, (**b**) cross-sectional features, (**c**) AFM images.

Combined modification with DDP and CA/PEI intermediate layers greatly modifies the structural features (surface shape, thickness and roughness) of PA layers, as visualized in Figure 6. Without any additive or interlayer, the conventional t-IP membrane synthesized by pure PIP-TMC reaction shows the classic nodular protrusions widely observed on semi-aromatic polyamide membranes. This distinctive surface feature arises from interfacial fluctuations induced by heat release during monomer cross-coupling, which accelerates regional polymerization rates unevenly [35,36]. Meanwhile, the t-IP membrane features an ultrathin PA layer with a thickness of 119 ± 10 nm. Due to this insufficient coating thickness, it fails to fully cover the bumpy surface and pore recesses on the large-pore PES substrate. Consequently, this membrane delivers the largest arithmetic average roughness (Ra = 45.9 ± 0.5 nm) in the whole group. After incorporating DDP, the surfaces of the SAIP and SIAIP membranes become much smoother, and the original distinct macroporous structures disappear completely. This demonstrates that DDP located at the water–oil interface effectively improves interfacial stability during IP and enables a more uniform polymerization process [37]. Furthermore, adding DDP surfactant greatly raises the thickness of PA layers for SAIP and SIAIP membranes. This morphological change can be explained by the fact that DDP reduces the surface tension of the interface, promoting the diffusion of amine monomers. More amine monomers infiltrate the newly formed PA layer and sustain steady diffusion toward the organic side. Consequently, alleviating the self-limiting nature of IP gives rise to a thicker PA layer and a lower surface roughness [38].

Slight differences in surface morphology are observed between the SAIP and SIAIP membranes. The SIAIP membrane shows ridge-like features. Although such structures are not fully developed, obvious protrusions can be seen on the surface, which tend to interconnect and form continuous ridges [28]. Surface wettability of underlying support largely governs the development of this morphology. The aqueous PIP solution tends to form discrete aqueous microdomains on the modified PES-CA/PEI substrate. These microdomains act as aqueous templates during IP, facilitating the generation of ridge-like structures on PA layer [39].

### 3.4. Surface Properties of Membranes

**Figure 7:**
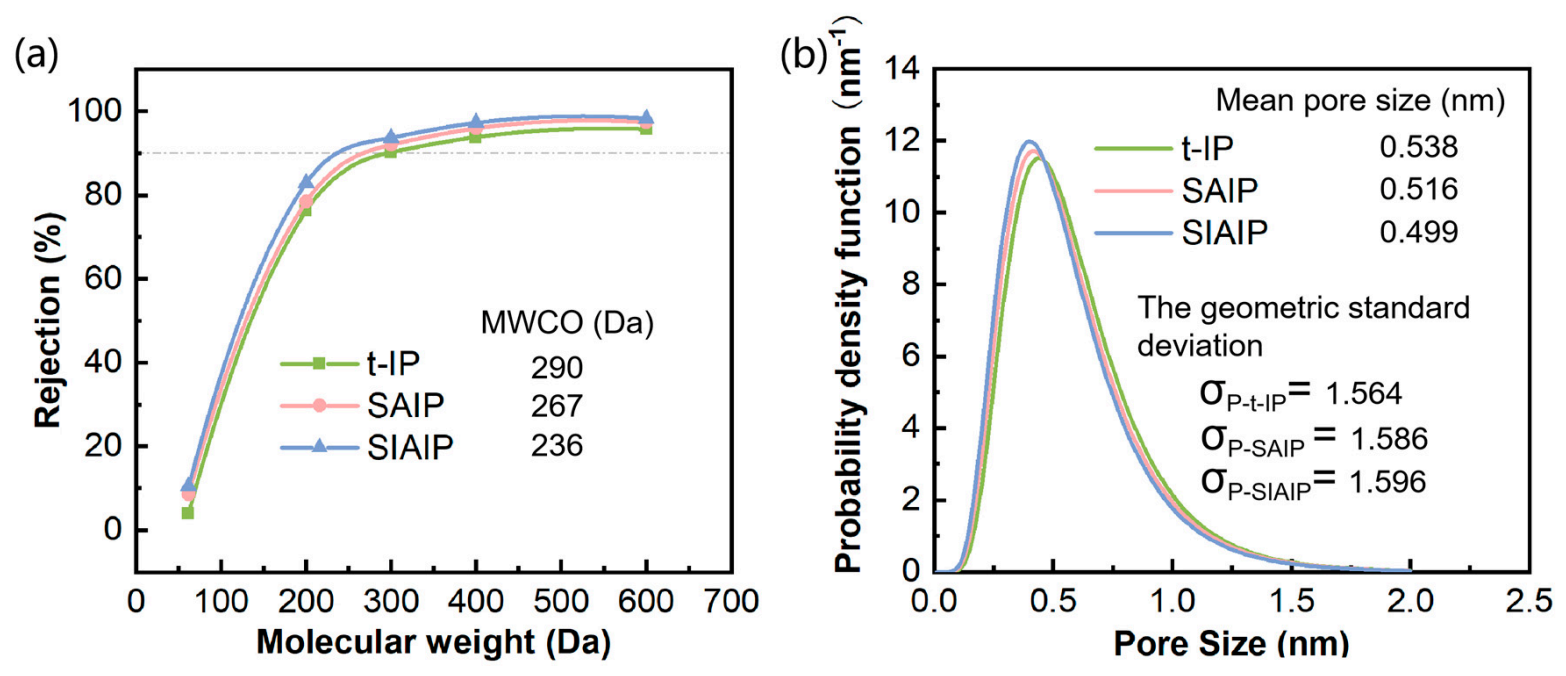
(**a**) MWCO and (**b**) pore size distribution of prepared membranes.

Neutral solute transport method [40] was employed to estimate the molecular weight cut-off (MWCO) of fabricated membranes. Meanwhile, the distribution range and average value of pore size were derived from the same data. Detailed calculation procedures are available in Supporting Information Section S4 [41,42]. Ion selectivity in PA NF membranes arises from a synergistic interplay of hydrodynamic filtration (steric hindrance), solution-diffusion, and Donnan exclusion. Accordingly, membrane rejection performance is primarily dictated by the uniformity of the nanopore structure, the physicochemical nature of the selective layer, and the density and sign of surface charge [43,44]. As shown in Figure 7, the highest MWCO (290 Da) and the largest mean pore size (0.538 nm) are both observed for the t-IP membrane. The phenomenon can be explained by the characteristics of traditional IP reactions: PIP monomers diffuse quickly within the bulk water solution, while their migration through the oil–water interface proceeds slowly and in a disordered manner. The inadequate replenishment of PIP monomers cannot compensate for their continuous consumption during the polymerization process, which leads to inhomogeneous microstructures and low densification. Compared with the t-IP membrane, the SAIP membrane possesses a markedly smaller mean pore size, which drops to 0.516 nm as a result of DDP incorporation. The underlying reason is that during IP, DDP can form a dense molecular layer at water–oil interface. Via electrostatic interactions between its phosphate groups and the amino groups of PIP molecules, DDP directionally adsorbs PIP at two-phase interface, remarkably accelerating interfacial migration, which facilitates the construction of dense PA separation films [24].

The measured pore sizes of SAIP membranes seem to be inconsistent with the crosslinking degree analyzed in Section 3.2. In fact, XPS typically samples a depth of about 10 nm, while SEM reveals that all three membranes possess PA layers with thicknesses exceeding 100 nm. Hence, the crosslinking degree calculated from O/N atomic ratios only reflects the condition of outermost PA layer, instead of interior properties [45]. On this basis, we infer that the SAIP membrane possesses a gradient crosslinking structure, namely a loose top layer and a relatively dense interior. The formation mechanism is proposed as follows: PIP undergoes unconfined free diffusion in bulk aqueous phase, and diffusion-promoting effect of DDP at the interface drives abundant PIP molecules to migrate into deeper regions of the PA layer. Meanwhile, PIP cannot be replenished quickly at the interface, ultimately forming a loosely structured top layer with low crosslinking degree [26]. Compared with SAIP membranes, the SIAIP membrane has reduced mean pore size (0.499 nm). This improvement is mainly attributed to the CA/PEI interlayer, which is conducive to the storage of amine monomers, facilitating the reaction and thereby increasing the crosslinking degree. Consequently, a PA separation layer featuring excellent integrity and high densification is successfully constructed.

**Figure 8:**
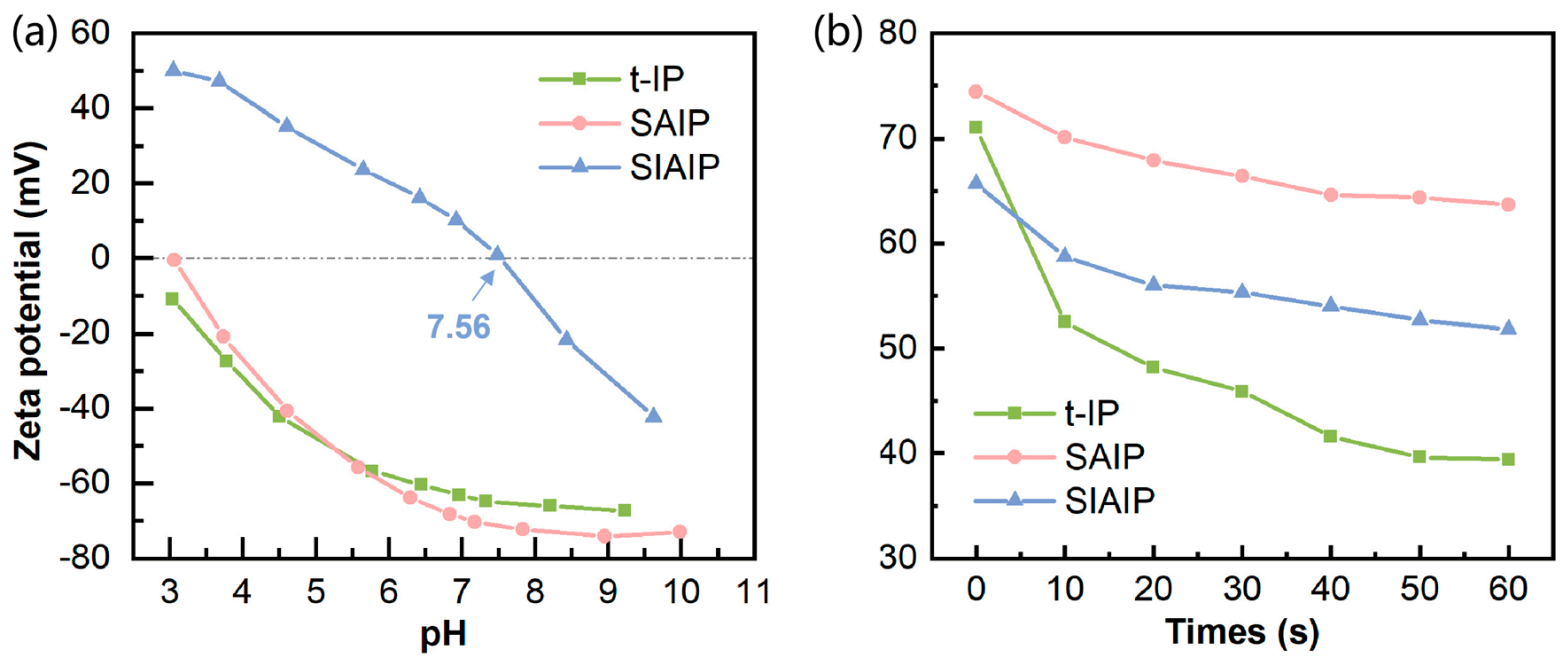
(**a**) Zeta potential and (**b**) dynamic WCA of fabricated membranes.

Figure 8a provides the zeta potential measurements of membrane surface charges, which were obtained via a zeta analyzer. Both t-IP and SAIP membranes naturally carry negatively charged surfaces, a feature originating mainly from the hydrolysis of acyl chloride moieties that were not consumed in the IP. Nevertheless, introducing a CA/PEI intermediate layer triggers full surface charge reversal on SIAIP membranes, whose isoelectric point is measured to be pH 7.56. Such a dramatic shift in surface electrical state fundamentally stems from abundant positively charged sites supplied by the bottom CA/PEI supporting interlayer. The CA/PEI interlayer possesses strong intrinsic positive charges, which effectively modulates the charge characteristics of the resultant PA layer. This phenomenon has been verified by the relevant literature [46,47]. In summary, the synergistic effect of DDP combined with the CA/PEI intermediate layer can effectively shrink the pore diameter and upregulate the surface positive charge of SIAIP membranes. The simultaneous optimization of membrane pore structure and surface electrical characteristics strengthens both steric sieving and Donnan exclusion effects, benefiting the improvement of Li^+^/Mg^2+^ selectivity. Figure 8b shows the WCA of different NF membranes. The WCA value is strongly influenced by both surface free energy and topographical roughness of solid materials [48]. The t-IP membrane presents lowest WCA and the highest surface hydrophilicity. This can be explained by the Wenzel equation, as the t-IP membrane has the largest surface roughness [49]. Notably, the SIAIP membrane is rich in hydrophilic amino groups. Despite its lowest surface roughness, it exhibits better hydrophilicity than the SAIP membrane, indicating that hydrophilic functional groups are key factors affecting membrane surface wettability.

### 3.5. Permeation and Li^+^/Mg^2+^ Selectivity of Membranes

To determine optimal fabrication conditions for the SAIP membrane, the effect of DDP dosage on permeability and separation performance was systematically investigated with fixed concentrations of PIP (0.5 wt%) and TMC (0.05 wt%). 0.15 mM was selected as the optimal DDP dosage. The detailed investigation on the optimal fabrication conditions for the SAIP membrane is presented in Supporting Information Section S5 and Figure S4.

**Figure 9:**
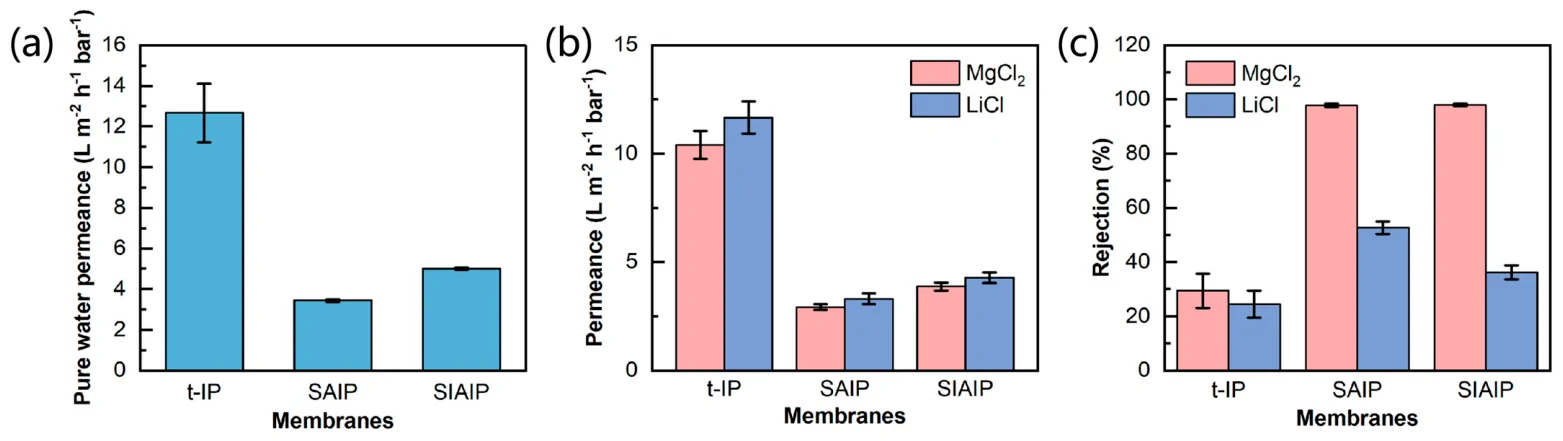
(**a**) PWP, (**b**) MgCl_2_ and LiCl permeability, (**c**) MgCl_2_ and LiCl rejection of three NF membranes.

Under optimal preparation parameters for the CA/PEI interlayer and the optimal DDP dosage obtained above, the test results of three as-fabricated NF membranes are presented in Figure 9. Owing to increased PA layer thickness and reduced surface hydrophilicity caused by DDP incorporation, the permeance of SAIP membrane decreases by 72.8% compared with the t-IP membrane. Owing to decreased average pore size, the size-sieving capability is strengthened, thereby enhancing the MgCl_2_ rejection of SAIP membrane from 29.4% to 97.8% and LiCl from 24.4% to 52.7%, indicating a greatly improved rejection capability toward Mg^2+^. Introducing the hydrophilic CA/PEI intermediate layer raises the pure water permeance (PWP) of SIAIP membrane by 31.3% relative to the SAIP counterpart. This improvement arises from the formation of thinner polyamide films and well-tuned water diffusion pathways within the membrane matrix. Moreover, the complete switch of surface electrical property from negative to positive greatly intensifies Donnan repulsion force. Such strengthened electrostatic repulsive interaction can well compensate for crosslinking imperfections induced by the hydrophilic underlayer, thereby sustaining a steady MgCl_2_ rejection efficiency of 97.9%. Meanwhile, the retention rate toward LiCl declines from 52.7% to 36.2%. This phenomenon is explained by the “drainage channel effect” derived from the CA/PEI interlayer, which optimizes the transport pathways of water molecules and Li^+^ and substantially reduces the transmembrane transport resistance of Li^+^. Overall, benefiting from the synergistic effect of DDP and CA/PEI, the SIAIP membrane achieves simultaneous improvements in size-sieving and Donnan effects, thus exhibiting excellent Li^+^/Mg^2+^ separation performance.

**Figure 10:**
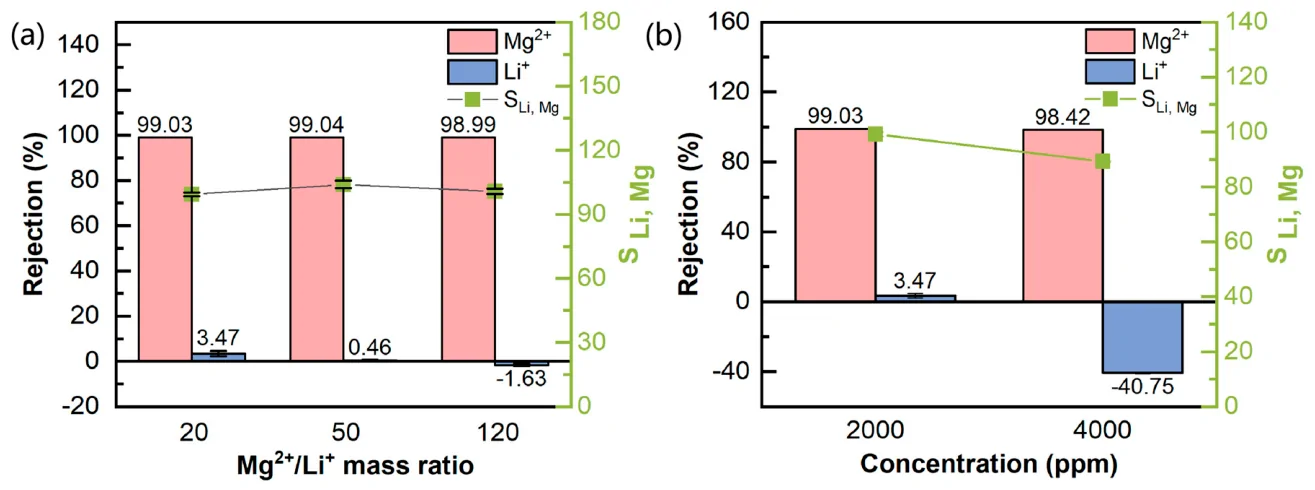
Mg^2+^/Li^+^ rejection rates and *S_Li_*_,*Mg*_ of SIAIP membrane in mixed MgCl_2_/LiCl solution under (**a**) different MLR and (**b**) different feed concentrations.

Single-salt perm-selectivity measurements are limited to characterizing ion transport across the membrane. Yet, for real-world lithium recovery from salt lakes, NF membranes must demonstrate robust separation ability under multi-salt mixed environments. Accordingly, systematic characterizations on Li^+^/Mg^2+^ separation behavior of SIAIP membrane were performed with various magnesium–lithium ratios (MLR) (total salt concentration: 2000 ppm; MLR = 20, 50, and 120) and different feed concentrations (MLR = 20; total salt concentration = 2000 ppm and 4000 ppm). Results are displayed in Figure 10. With MLR increasing, the Mg^2+^ rejection capacity of the SIAIP membrane first rose and then declined. Meanwhile, the Li^+^/Mg^2+^ selectivity factor (*S_Li_*_,*Mg*_) reached a peak of 104.0 after starting at 99.3, and eventually settled at 100.7. This variation trend is consistent with findings reported in the previous literature [50]. Concentration polarization accounted for the upward variation in *S_Li_*_,*Mg*_ at low MLR. When the system operated under low MLR (≤60), massive Mg^2+^ ions accumulated on the membrane surface, generating a localized ion concentration far higher than that in bulk feed solution. Such obvious concentration gradients prompted Mg^2+^ to diffuse backward toward the feed. This process simultaneously resulted in higher Mg^2+^ rejection and an improved *S_Li_*_,*Mg*_ [9,51]. Elevating MLR to higher level (≥80) brought about a dramatic growth of osmotic pressure difference across the membrane. The strong osmotic gradient facilitated the movement of Mg^2+^ across the membrane, lowering its rejection and overall separation factor [52]. Raising the mixed-salt total concentration from 2000 ppm to 4000 ppm produced a parallel decrease in both Mg^2+^ and Li^+^ rejections, as well as a decrease in the *S_Li_*_,*Mg*_, which dropped to 89.2 from 99.3. This phenomenon is ascribed to the increased Cl^−^ ionic strength under higher feed concentrations. This enhanced ionic strength amplifies the charge-screening action at the membrane surface while suppressing Donnan exclusion, which in turn leads to a decline in Mg^2+^ rejection [53,54]. Moreover, rising salinity contributes to higher osmotic pressure in the system, as well as to more severe concentration polarization. Consequently, these combined effects lead to a decline in water permeability and simultaneously degrade the capacity of membrane to reject ions [52]. Notably, negative rejection [55,56,57] of Li^+^ was observed during the tests, which was primarily governed by the migration kinetics of Cl^−^. Cl^−^ passed through the membrane with extremely low mass transfer resistance. For the whole solution to remain electrically neutral, Li^+^ was obliged to transport alongside Cl^−^. Such coupled ion diffusion rendered higher lithium concentration in permeate than that present in initial feed solution, which further produced negative retention coefficients. This unique characteristic in multi-component salt filtration turned out to be beneficial, as it greatly improved the separation selectivity for Li^+^ over Mg^2+^ of the membrane [58]. In summary, the SIAIP membrane exhibits favorable separation stability over a wide MLR range, whereas its adaptability to high-concentration mixed salt solutions is limited.

**Figure 11:**
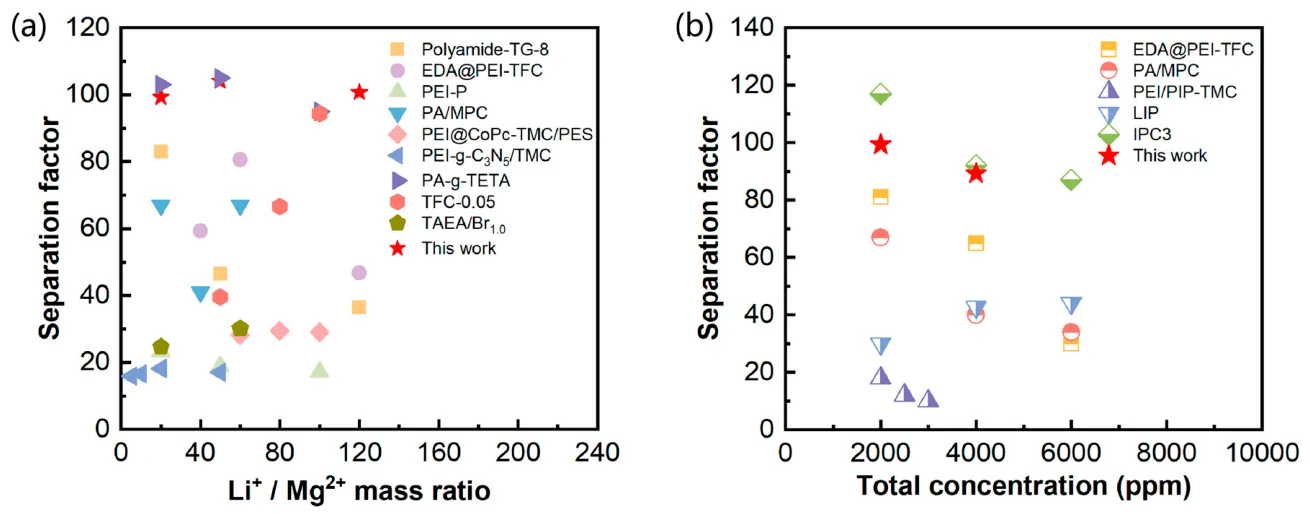
Comparison of Li^+^/Mg^2+^ selectivity between SIAIP membranes and reported membranes under (**a**) different MLRs and (**b**) different feed concentrations.

To underscore the high Li^+^/Mg^2+^ selectivity of the SIAIP membrane we prepared, we contrasted its *S_Li_*_,*Mg*_, measured under diverse MLR and mixed-salt conditions with values of literature-reported NF membranes. The relevant results are presented in Figure 11, Table 2 and Table 3. Although the SIAIP membrane does not possess the highest separation factor, it exhibits superior operational stability across different MLR and salt concentration conditions. Such benefits stem from its smaller average pores and tighter pore size distribution. Unlike common positively charged nanofiltration membranes that merely rely on Donnan repulsion to separate different ionic species, the SIAIP membrane takes advantage of synergistic interactions between DDP additives and the CA/PEI interlayer. The joint regulation strengthens steric sieving and electrostatic repulsion simultaneously. Such dual working principles efficiently weaken charge shielding effects commonly generated by Mg^2+^ enrichment near the membrane surface boundary.

## 4. Conclusions

In summary, utilizing the combined effect of surfactants and interlayers, dual-charged NF membranes are fabricated via the SIAIP strategy in this work to achieve selective Li^+^/Mg^2+^ separation. We systematically explore how the fabrication parameters of the CA/PEI interlayer and DDP dosage influence the permeability and solute-rejection behaviors of resulting NF membranes, as well as the relevant working mechanisms. The joint modulation by the CA/PEI interlayer and DDP enables simultaneous enhancement of both size-sieving and Donnan repulsion. The optimized SIAIP membrane delivers 97.9% MgCl_2_ rejection and 36.2% LiCl rejection, with a Li^+^/Mg^2+^ separation factor of 99.3. Owing to its narrow pore size distribution and low surface roughness, the membrane is less susceptible to electrostatic screening and concentration polarization in mixed salt solutions. When compared with other conventional pressure-driven NF membranes under varied MLR and feed concentrations, the SIAIP membrane presents outstanding operational stability and adaptability. In conclusion, the SIAIP membrane developed in this work combines excellent Li^+^/Mg^2+^ separation performance and favorable adaptability to varied operating conditions, demonstrating tremendous feasibility for practical utilization in Li recovery from brine sources in salt lakes.

## Figures and Tables

**Table 1:** Fabrication parameters of different NF membranes.

Samples	NaIO_4_ Concentration (wt%)	CA Concentration (wt%)	PEI Concentration (wt%)	DDP Addition (mM)	PIP Concentration (wt%)	TMC Concentration (wt%)
t-IP	0	/	/	/	0.5	0.05
SAIP	0	/	/	0.5	0.5	0.05
SIAIP	0.05	1	0.3	0.5	0.5	0.05

**Table 2:** Experimental conditions of SIAIP membrane and other reported pressure-driven membranes in mixed salt solutions with different MLRs.

Number	Membrane	Mg^2+^/Li^+^ Mass Ratio (Feed Solution)	Separation Factor	Testing Concentration	Testing Pressure	Testing Membrane Area	Reference
1	Polyamide-TG-8	20	82.96	2000 ppm	4 bar	1.54 cm^2^	[59]
		50	46.40				
		120	36.40				
2	EDA@PEI-TFC	40	59.20	2000 ppm	4 bar	4.9 cm^2^	[20]
		60	80.62				
		120	46.79				
3	PEI-P	20	23.3	2000 ppm	6 bar	7.1 cm^2^	[60]
		50	18.9				
		100	17.2				
4	PA/MPC	20	67	2000 ppm	5 bar	7.06 cm^2^	[61]
		40	41				
		60	67				
5	PA-g-TETA	20	103	2000 ppm	-	-	[62]
		50	105				
		100	95				
6	PEI@CoPc-TMC/PES	60	28.2	2000 ppm	2 bar	28.7 cm^2^	[63]
		80	29.5				
		100	29.0				
7	TFC-0.05	20	33.38	2000 ppm	6 bar	22.051 cm^2^	[64]
		50	39.56				
		80	66.63				
		100	94.28				
8	TAEA/Br1.0	20	24.6	2000 ppm	2 bar	23.0 cm^2^	[50]
		60	30.1				
9	PEI-g-C_3_N_5_/TMC	5	15.9	2000 ppm	6 bar	10.18 cm^2^	[65]
		10	16.62				
		20	18.18				
		50	17.09				
10	SIAIP	20	99.26	2000 ppm	5 bar	22.05 cm^2^	This work
		50	104.02				
		120	100.71				

**Table 3:** Experimental conditions of SIAIP-DDP membrane and other reported pressure-driven membranes in mixed salt solutions of different concentrations.

Number	Membrane	Mg^2+^/Li^+^ Mass Ratio (Feed Solution)	Separation Factor	Testing Concentration	Testing Pressure	Testing Membrane Area	Reference
1	IPC33	20	117	2000 ppm	10 bar	7.06 cm^2^	[66]
			92	4000 ppm			
			87	6000 ppm			
2	EDA@PEI-TFC	60	81	2000 ppm	4 bar	4.9 cm^2^	[20]
			65	4000 ppm			
			30	6000 ppm			
3	PA/MPC	20	67	2000 ppm	5 bar	7.06 cm^2^	[61]
			40	4000 ppm			
			34	6000 ppm			
4	PEI/PIP-TMC	30	18	2000 ppm	2 bar	12.56 cm^2^	[67]
			12	2500 ppm			
			10	3000 ppm			
5	LIP	20	29.9	2000 ppm	10 bar	7.06 cm^2^	[68]
			42.7	4000 ppm			
			44.1	6000 ppm			
6	SIAIP	20	99.26	2000 ppm	5 bar	22.05 cm^2^	This work
			89.23	4000 ppm			

## Data Availability

Dataset available on request from the authors.

## Supplementary Materials

The following supporting information can be downloaded at: https://www.mdpi.com/article/10.3390/membranes16080270/s1, Figure S1: The (a) PWP, (b) MgCl_2_ and LiCl permeance, (c) MgCl_2_ and LiCl rejection of membranes prepared at different CA concentrations; Figure S2: The (a) PWP, (b) MgCl_2_ and LiCl permeance, (c) MgCl_2_ and LiCl rejection of membranes prepared at different PEI concentrations; Figure S3: The (a) PWP, (b) MgCl_2_ and LiCl permeance, (c) MgCl_2_ and LiCl rejection of membranes prepared under different deposition times; Figure S4: The (a) PWP, (b) MgCl_2_ and LiCl permeance, (c) MgCl_2_ and LiCl rejection of membranes prepared at different DDP concentrations; Table S1: XPS results of three types of NF membranes.
