# Supplementary material for: Positively Charged NF Membranes with Co-Enhanced Donnan and Size-Sieving Effects Fabricated Toward Efficient Li+/Mg2+ Separation
Source: Membranes (Basel). 2026 Aug 13;16(8):270. doi: 10.3390/membranes16080270 (PMC13515611; doi:10.3390/membranes16080270)
Supplement: Supplementary file 1 [file membranes-16-00270-s001.zip › membranes-4453047-supplementary.pdf]

Positively Charged NF Membranes with Co-Enhanced Donnan and Size-Sieving Effects Fabricated Toward Efficient Li^+/Mg^{2+} Separation

Yu-Tong Yin [1], Rui Jia [1], Zhen-Liang Xu [1,2,*], Sen Wang [1] and Rui Han [1]

1 State Key Laboratory of Chemical Engineering and Low-Carbon Technology, Membrane Science and Engineering R&D Lab, Chemical Engineering Research Center, School of Chemical Engineering, East China University of Science and Technology, 130 Meilong Road, Shanghai 200237, China

2 Shanghai Electronic Chemicals Innovation Institute, East China University of Science and Technology, Shanghai 200237, China

* *Correspondence: chemxuzl@ecust.edu.cn; Tel.: +86-21-6425-3670; Fax: +86-21-6425-2989*

This file includes:

1. Characterization and membrane performance test

2. Optimal preparation conditions for the CA/PEI intermediate layer

3. Crosslinking degree of the polyamide membranes

4. Molecular weight cut-off (MWCO) and pore size distribution

5. Optimal fabrication conditions for the SAIP membrane

Supplementary Figures S1-4

Supplementary Table S1

Supplementary References 1-3

1. Characterization and membrane performance test

The surface and cross-sectional morphologies of NF membranes were characterized by SEM (Nova Nano SEM450). Membrane specimens were cut into small pieces and mounted face-up for surface observation. To obtain cross-sectional images, membranes were fractured in liquid nitrogen and fixed with the fractured surface outward. All samples were sputter-coated with platinum for 90 s to enhance conductivity.

Surface topography and roughness parameters of the NF membranes were characterized using atomic force microscopy (AFM, Bruker Dimension Icon). The instrument detects microscale surface undulations via interactive forces between the probe tip and surface atoms, and converts probe displacement signals into three-dimensional topographic images. For testing, flat-sheet membrane specimens were trimmed to suitable dimensions and immobilized on silicon wafers. The scanning area was set as 10 μm×10 μm.

Attenuated total reflectance Fourier-transform infrared spectroscopy (ATR-FTIR, Thermo Scientific Nicolet iS20) was employed to characterize the surface of NF membranes. Infrared radiation was irradiated onto the membrane surface, and the absorption and transmission signals at different wavenumbers were recorded to generate characteristic spectra for analyzing the types of functional groups and chemical composition on the membrane surface. Spectra were acquired over the wavenumber range of 400-4000 cm-1.

X-ray photoelectron spectroscopy (XPS, Thermo Scientific K-Alpha+) was

utilized to analyze the elemental composition of the membrane surface based on the photoelectric effect. X-ray irradiation excites surface atoms to emit photoelectrons. The photoelectron energy signals are acquired for the analysis of binding energies and signal intensities. The typical probing depth of XPS is below 10 nm. For data processing, the C 1s peak at 284.8 eV was adopted for charge correction.

The hydrophilicity of NF membranes was evaluated via water contact angle meter (WCA, JC2000A). A fixed volume of deionized water was placed on the membrane surface. The dynamic evolution of droplets was recorded by a CCD camera within 60 s at an interval of 10 s. At least three separate regions of each specimen were tested for reproducibility, and the average of repeated measurements was presented as the final result.

The surface charge properties of NF membranes were characterized using a streaming potential analyzer (SurPASS 3, Anton Paar) to determine zeta potential via the streaming potential technique. Measurements were performed using 1 mmol/L KCl aqueous solution as the electrolyte, and the pH of the testing system was adjusted from 3 to 10.

The permeation and separation performance of the NF membrane was tested using a laboratory-made cross-flow filtration device with the test area of 22.05 cm^2 and the crossflow velocity of 22 $cm \cdot s^{-1}$. The temperature was kept at 25 ºC, and the membrane should be pre-pressurized at 5 bar for 30 min before the test. The concentration of the three inorganic salt ($MgCl_2$, LiCl, and Na_2SO_4) solutions was 1000 ppm, and the salt concentrations in the feed and permeate solutions were analyzed by a

conductivity meter (Leichi, DDS-11A). For accuracy, the mean and error bars were calculated using data from at least three sets of parallel experiments under the identical conditions.

The membrane permeance was calculated as Eq. (1):

$$J = \frac{V}{A \times \Delta t \times P} \tag{1}$$

Where V (L) denoted the permeate volume, A (m^2) referred to the filtration area, Δt (h) represented the duration of the filtration process, and P (bar) indicated the applied pressure.

The rejection (R, %) of inorganic salts was obtained by Eq. (2):

$$R = (1 - \frac{C_p}{C_f}) \times 100\% \tag{2}$$

Where C_p and C_f correspond to the salt concentrations in the permeate and feed solutions, respectively (g L^{-1}).

Mixed salt solutions of $MgCl_2$/LiCl with varying magnesium-lithium ratios (20, 50, 120) and concentrations (2000 ppm, 4000 ppm) were prepared to evaluate the Li^+/Mg^{2+} selectivity of the NF membranes. The Li^+/Mg^{2+} selectivity factor ($S_{Li, Mg}$) was subsequently calculated using the Eq. (3):

$$S_{Li,Mg} = \frac{C_{Li^+,p}/C_{Mg^{2+},p}}{C_{Li^+,f}/C_{Mg^{2+},f}} \tag{3}$$

where $C_{Li^+,f}, C_{Mg^{2+},f}$ and $C_{Li^+,p}, C_{Mg^{2+},p}$ were the Li^+ and Mg^{2+} concentrations before and after membrane filtration. The ion concentrations in the mixed salt solution were analyzed using an inductively coupled plasma mass spectrometer (ICP, Agilent 5110-OES).

2. Optimal preparation conditions for the CA/PEI intermediate layer

With the DDP dosage fixed at 0.15 mM, the effects of CA concentration, PEI concentration, and deposition time on the performance of the resultant NF membranes were investigated, and the results are displayed in **Figs. S1-S3**. When the PEI concentration was kept at 0.3 wt% and the deposition time was set to 20 min, the pure water permeance (PWP) of the membranes increased gradually with the rise of CA concentration and reached the maximum at a CA concentration of 1 wt%. Notably, varying CA concentration exerted no obvious influence on the $MgCl_2$ rejection, which remained above 97% throughout the tests. In contrast, CA concentration significantly affected the LiCl rejection of NF membranes. As the CA concentration increased from 0.3 wt% to 1 wt%, the LiCl rejection dropped sharply from 53.4% to 36.2%. The decrease in LiCl rejection corresponded to the increase in pure water permeance, which complied with the typical perm-selectivity trade-off effect in membrane separation. Overall, the NF membrane achieved the optimal combination of separation and permeance at a CA concentration of 1 wt%. Accordingly, 1 wt% was selected as the optimal CA concentration for membrane fabrication.

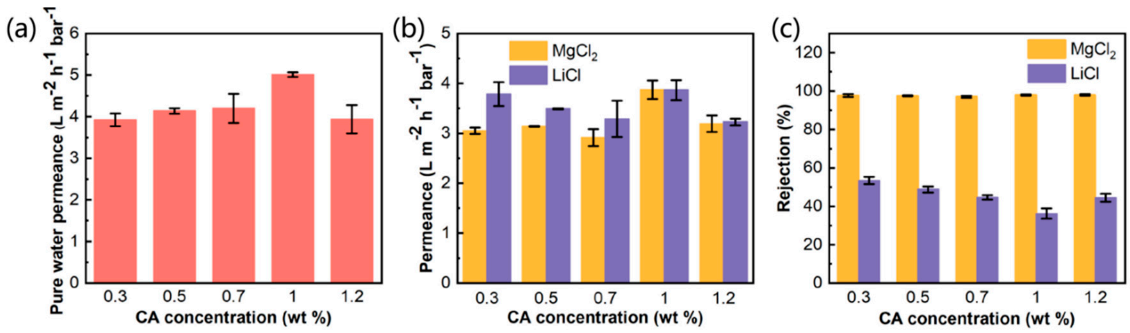

Figure S1. The (a) PWP, (b) $MgCl_2$ and LiCl permeance, (c) $MgCl_2$ and LiCl rejection of membranes prepared at different CA concentrations.

PEI concentration exerts a remarkable effect on the $MgCl_2$ rejection performance of NF membranes. Experimental results show that the $MgCl_2$ rejection is only 95.5% at a PEI concentration of 0.1 wt%. When the PEI concentration increases to 0.3 wt%, the $MgCl_2$ rejection rises markedly to 97.9%. Nevertheless, further increasing the PEI concentration to 0.5 wt% fails to achieve a continuous improvement, and the $MgCl_2$ rejection declines to 95.1% instead. It can be concluded that a moderate increase in PEI concentration modulates the surface charge properties of the membrane and thus effectively enhances the rejection of divalent cations (Mg^{2+}). However, excessively high PEI concentration imposes adverse impacts on separation performance. This phenomenon is probably attributed to the formation of defects in the PA layer caused by an overly thick CA/PEI interlayer. Based on the above results, the optimal PEI concentration is determined to be 0.3 wt%.

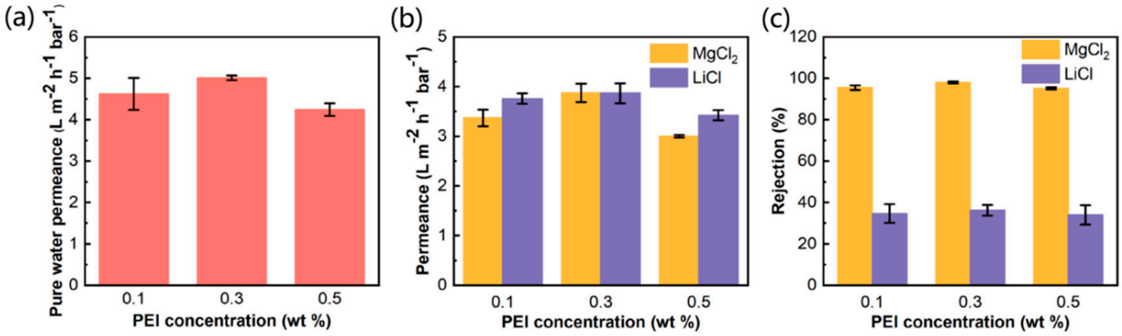

Figure S2. The (a) PWP, (b) $MgCl_2$ and LiCl permeance, (c) $MgCl_2$ and LiCl rejection of membranes prepared at different PEI concentrations.

Prolonged deposition time increases the thickness of the interlayer, which raises the proportion of water transport within the hydrophilic interlayer and ultimately accelerates the transport rate of water molecules. Accordingly, the pure water permeability and single salt solution permeance of the membranes generally increase

with extended deposition time. Nevertheless, an excessively thick hydrophilic interlayer tends to induce structural defects in the PA layer, leading to a decline in ion rejection. When the deposition time was prolonged from 20 min to 40 min, the rejection of $MgCl_2$ dropped sharply from 97.9% to 96.0%. Considering the comprehensive effects of deposition time on membrane permeance and rejection performance, the optimal deposition time for the interlayer was determined to be 20 min.

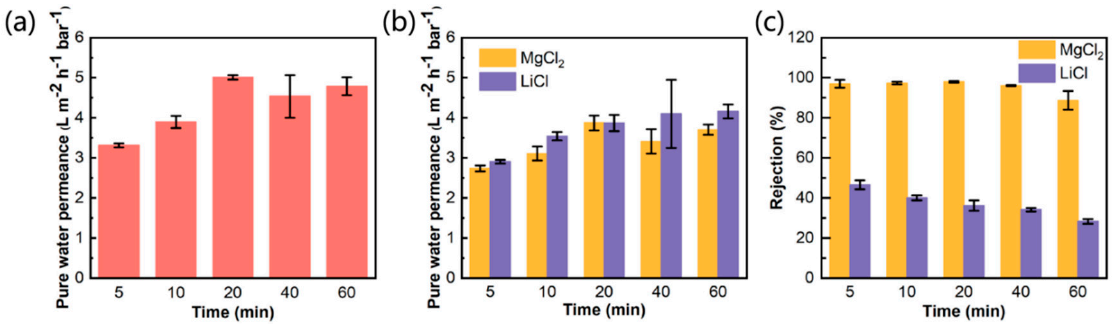

Figure S3. The (a) PWP, (b) $MgCl_2$ and LiCl permeance, (c) $MgCl_2$ and LiCl rejection of membranes prepared under different deposition times.

3. Crosslinking degree of the polyamide membranes

The network crosslinking degree of polyamide NF membranes, formed through the reaction between PIP and TMC, can be determined using Eq. (4) and (5) [1]:

$$DNC = \frac{X}{X+Y} \times 100\% \quad (4)$$

$$\frac{O}{N} = \frac{3X+4Y}{3X+2Y} \quad (5)$$

The O/N ratio value can be obtained from the XPS spectra. *X* and *Y* represented the fractions of fully networked and fully linearly crosslinked polyamide, respectively. The elemental ratios and cross-linking degrees of the three NF membranes were listed in **Table S1**.

Table S1. XPS results of three types of NF membranes

Sample	Surface element content (%)			O/N	Crosslinking degree (%)
	C (%)	N (%)	O (%)		
t-IP	71.93	11.71	16.36	1.40	50.0
SAIP	73.80	10.06	16.14	1.60	30.7
SIAIP	70.92	12.45	16.63	1.34	56.4

4. Molecular weight cut-off (MWCO) and pore size distribution

The MWCO properties were acquired by testing the rejection of ethylene glycol (Mn = 62) and polyethylene glycol (Mn = 200, 300, 400, 600) at a concentration of 200 ppm, and MWCO was defined as the molecular weight of the PEG at the rejection rate of 90%. The solute concentrations in the feed and permeate solutions were measured by a total organic carbon analyzer (TOC, Shimadzu) [2].

The pore size distribution was determined by the solute transport method. Eq. (6) illustrates the connection between the solute molecular radius (r_s) and the solute rejection rate (R) [3].

$$F(R) = A + Bln\, r_s \tag{6}$$

The Stokes radius (r_s, m) of the PEG molecules can be calculated using Eq. (7).

$$r_s = 16.73 \times 10^{-12} \times MW^{0.557} \tag{7}$$

The pore size distribution of the NF membranes can be estimated using the probability density function (Eq. (8)).

$$\frac{dR(d_p)}{dd_p} = \frac{1}{d_p ln\, \sigma_p \sqrt{2\pi}} exp[-\frac{(ln\, d_p - ln\, \mu_p)^2}{2(ln\, \sigma_p)^2}] \tag{8}$$

where d_p denotes the pore size, σ_p represents the geometric standard deviation of the probability density function curve, and μ_p indicates the mean pore size of membranes.

5. Optimal fabrication conditions for the SAIP membrane

To determine the optimal fabrication conditions for the SAIP membrane, the effect of DDP dosage on the permeability and separation performance of NF membranes was systematically investigated with fixed PIP concentration (0.5 wt%) and TMC concentration (0.05 wt%). The corresponding results are shown in **Fig. S4**. As the DDP dosage increased, both PWP and salt solution flux of the membranes decreased obviously, while the rejection rate rose remarkably. This is because excessive DDP accumulates at the water-oil interface and accelerates the diffusion of amine monomers, which further increases the crosslinking degree of the PA separation layer. When the DDP concentration increased from 0.1 mM to 0.15 mM, the rejection of $MgCl_2$ rose sharply from 93.5% to 97.8%, and the rejection of LiCl increased from 45.9% to 52.7%. The membrane achieved the best separation performance in single-salt systems at this concentration. Accordingly, 0.15 mM was selected as the optimal DDP dosage in this work.

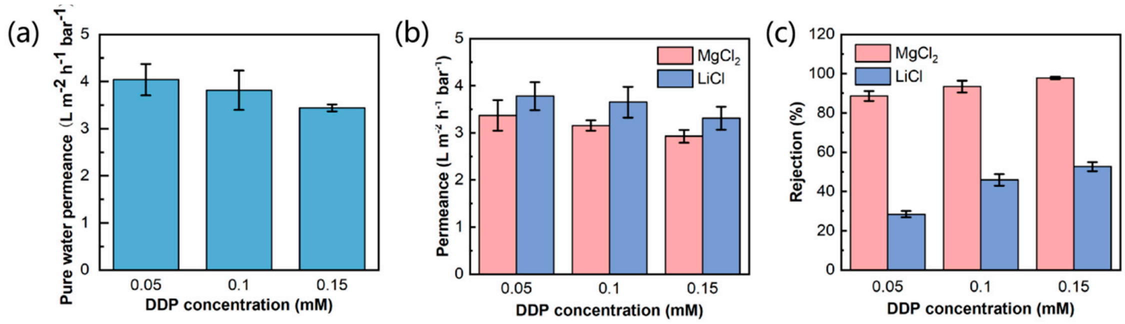

Figure S4. The (a) PWP, (b) $MgCl_2$ and LiCl permeance, (c) $MgCl_2$ and LiCl rejection of membranes prepared at different DDP concentrations

References

1. Karan, S.; Jiang, Z.; Livingston, A.G. Sub-10 nm polyamide nanofilms with ultrafast solvent transport for molecular separation. *Science* **2015**, doi:10.1126/science.aaa5058.
2. Chen, K.; Li, F.; Wei, T.; Zhou, H.; Zhang, T.; Zhao, S.; Xie, T.; Sun, H.; Li, P.; Niu, Q.J. An interlayer-based positive charge compensation strategy for the preparation of highly selective Mg^{2+}/Li^{+} separation nanofiltration membranes. *J. Membr. Sci.* **2023**, doi:10.1016/j.memsci.2023.121882.
3. Mao, X.; Xu, N.; Shi, X.; Wen, H.; Liu, C. Integration of charge repulsion and size exclusion effects into the skin layer matrix for enhanced Mg^{2+}/Li^{+} nanofiltration separation. *J. Membr. Sci.* **2024**, doi:10.1016/j.memsci.2024.123315.
